# Discharging Patients for Lack of Nurses

**Published:** 1921-06-18

**Authors:** Dyce Duckworth, Henry Leonard

**Affiliations:** Chairman of the Medical Board. The Royal Sea-Bathing Hospital, Margate.; Chairman of the Committee of Management. The Royal Sea-Bathing Hospital, Margate.


*To the Editor of* THE HOSPITAL.

### Discharging Patients for Lack of Nurses.

*To the Editor of* THE HOSPITAL.

SIR,—The shortage of trained nurses and probationers at this hospital is so great that the Committee earnestly seeks the aid of your columns in making it known that they will be compelled to discharge sixty patients suffering from surgical tuberculosis unless suitable applicants come forward immediately. These patients would have to return to their homes, as there is no institution available to which they can be sent. Moreover, there are 120 applicants waiting to come in. Eight trained nurses and twelve probationers are required. Good salaries are offered, and when the staff is completed the hours on duty will be reduced. Full particulars may be obtained from the Matron, R.S.B. Hospital, Margate.—Yours faithfully,

DYCE DUCKWORTH, Chairman of the Medical Board. HENRY LEONARD, Chairman of the Committee of Management.

The Royal Sea-Bathing Hospital,
Margate.

